# Evaluation of Summer Maize Water and Nitrogen Management Strategies Across Different Hydrological Years Using the DSSAT Model

**DOI:** 10.3390/plants15121777

**Published:** 2026-06-09

**Authors:** Shikai Gao, Yihao Liu, Pengcheng He, Aofeng He, Xiaochuan Chen, Xinru Liu, Xuewen Gong

**Affiliations:** School of Water Conservancy, North China University of Water Resources and Electric Power, Zhengzhou 450045, China; igaoshikai@163.com (S.G.); 13239066390@163.com (Y.L.); ihepengcheng@163.com (P.H.); 18790639808@163.com (A.H.); 17814605265@163.com (X.C.); iliuxinru@163.com (X.L.)

**Keywords:** summer maize, DSSAT model, water–nitrogen management, growth and development, hydrological year types

## Abstract

Summer maize (*Zea mays* L.) production on the North China Plain is highly dependent on variable seasonal rainfall, which increases the likelihood that inappropriate water and nitrogen allocation will cause yield fluctuations and ecological and environmental risks. Previous studies have mainly relied on single-site field comparisons or basic statistical evaluation methods, limiting the understanding of the dynamic response mechanisms of drought stress coupled with nitrogen application during the jointing and grain-filling stages. Based on field experiments conducted in 2024–2025, the DSSAT model was used to simulate aboveground dry matter accumulation (CWAM), grain yield, leaf area index (LAI), dry matter evapotranspiration productivity (DMPEM), and dry matter nitrogen productivity (DPNAM) of summer maize under different water–nitrogen treatments at different growth stages. Then, historical meteorological data for Henan Province from 2003 to 2023 were imported. The years were classified into three hydrological year types: wet years, normal years, and dry years. Subsequently, Principal Component Analysis (PCA), the TOPSIS method, and the Rank-Sum Ratio (RSR) method were employed to construct a multidimensional evaluation system for assessing water and nitrogen management strategies under different hydrological year types. The results showed that the nitrogen application rate had a significant regulatory effect on yield, DPNAM, and DMPEM. All three initially increased and then decreased as the nitrogen application rate rose, with the optimal performance observed under the normal nitrogen (N2) treatment. Under drought conditions during the same growth stage, the increase in the maximum yield under the N2 treatment was approximately 8.1% and 50% higher than that under the high-nitrogen (N1) and low-nitrogen (N3) treatments, respectively. Compared with drought during the grain-filling stage, drought during the jointing stage had a smaller negative effect on CWAM and LAI. A comprehensive evaluation with long-term meteorological data reflects that drought during the jointing stage combined with normal nitrogen (Q2) is the optimal water–nitrogen management strategy for wet years (with an RSR value of 0.994). The treatments of drought during the jointing stage combined with high nitrogen (Q1) and drought during the grain-filling stage combined with normal nitrogen (H2) reveal greater adaptability and favorable universality across different hydrological year types. The model’s reliability under various water–nitrogen coupling conditions was validated by integrating field experiments, DSSAT model simulations, and a multidimensional evaluation system. This study lays a scientific theoretical foundation for achieving high and stable yields in summer maize under different water–nitrogen coupling conditions and across various hydrological year scenarios.

## 1. Introduction

According to demographic projections, the global population will continue to grow, and agricultural production will need to increase by 60% by 2050 to meet the rapidly rising demand for food, feed, fiber, and fuel [1]. Cereal crops are essential for meeting global nutritional and energy needs [2]. As one of the three major cereal crops worldwide, maize is a major source of food, feed, and industrial raw materials [3]. The North China Plain (NCP), one of the main summer maize-producing regions in China, contributes 33% of national maize production [4]. Irrigation and nitrogen fertilizer are key determinants of maize production, and appropriate water and nitrogen management can significantly improve summer maize yield, water use efficiency, and nitrogen use efficiency [5]. However, summer maize production in the NCP relies mainly on seasonal rainfall, which is generally insufficient during critical growth stages. Supplementary irrigation is therefore required to achieve high and stable yields [6]. Meanwhile, inappropriate nitrogen fertilizer application may cause both agronomic and ecological risks, including increased production costs, yield reduction, and groundwater pollution [7]. Therefore, optimizing water and fertilizer management is essential for ensuring national food security and mitigating environmental pollution.

To increase maize yield and improve water and nitrogen use efficiency, various field management strategies have been proposed, including integrated water and nitrogen management, improved tillage practices, and high planting density [8]. Among them, integrated water and nitrogen management offers clear advantages for efficient resource use in agricultural systems [9]. Appropriate irrigation and nitrogen application rates can effectively increase soil available nitrogen and enzyme activity, thereby enhancing crop yield and nitrogen uptake [10]. Maize has a high water requirement, and water deficit can delay growth, reduce dry matter accumulation and nitrogen uptake, and ultimately result in substantial yield loss [11]. Under mild drought stress, however, a moderate increase in nitrogen application can enhance drought tolerance, improve nitrogen uptake, and promote yield formation [12]. Accordingly, regulating the water-to-fertilizer ratio at different developmental stages is critical for optimizing maize growth, achieving high and stable yields, and maximizing nitrogen uptake.

Crop models and field trials are two widely used approaches for optimizing irrigation and nitrogen fertilizer application [13]. Compared with field trials, crop simulation models account for the complex interactions among weather, soil, and management factors that affect crop performance, thereby effectively complementing field experiments [14]. Currently, used crop models include Agricultural Production Simulation Model (APSIM), AquaCrop, and Decision Support System for Agricultural Technology Transfer (DSSAT) [15]. However, these models differ substantially in simulation mechanisms and research focus. For example, APSIM performs well in crop rotation and intercropping systems, but may not consistently capture changes in dry matter yield and physiological photosynthetic processes at specific growth stages with sufficient accuracy [16]. AquaCrop is a water productivity model with strong robustness in simulating water stress and relatively low input requirements; nonetheless, it simplifies crop physiological processes and uses empirical fertilizer stress factors, allowing for reduced accuracy under nitrogen stress [17]. In contrast, DSSAT is based on a detailed daily growth mechanism and uses meteorological, soil, and field management data to accurately define crop growth stages and simulate the dynamic regulation of water and nitrogen on leaf area, dry matter allocation, and final yield across growth stages [18]. This model can describe the physiological processes of maize under different irrigation and nitrogen application regimes, supporting a more detailed analysis of water–nitrogen coupling mechanisms at different stages.

Water management and nitrogen fertilization are closely interrelated and interact significantly with maize nitrogen uptake, yield, and aboveground dry matter accumulation. Nevertheless, previous studies have mainly relied on model simulations or conventional statistical evaluations, and the dynamic response mechanisms associated with the coupling of nitrogen application and stage-specific water stress remain insufficiently understood. Additionally, summer maize production in the NCP is subject to considerable interannual variability in precipitation, and short-term trials alone typically make it difficult to develop management strategies with broad climatic adaptability. Therefore, a two-year field experiment was conducted, and the DSSAT model was calibrated and validated. Data on cumulative aboveground dry matter (CWAM), yield, leaf area index (LAI), dry matter evapotranspiration productivity (DMPEM), and dry matter nitrogen productivity (DPNAM) were comprehensively analyzed to clarify the effects of coupled water stress and nitrogen application rates on the growth and development of summer maize at different growth stages. After the DSSAT model parameters were calibrated and validated based on field trials, meteorological data from Henan Province for the period 2003–2023 were input into the model for a comprehensive evaluation through principal component analysis (PCA), the TOPSIS method, and the RSR method. Additionally, the optimal water and nitrogen management strategies were selected to achieve maximum crop yields and reinforce resource utilization efficiency, thereby providing a scientific foundation for the efficient utilization of agricultural resources and the achievement of high and stable grain production in Henan Province.

## 2. Pilot Zone, Experimental Design, and Model Performance

### 2.1. Overview of the Pilot Zone

**Figure 1:**
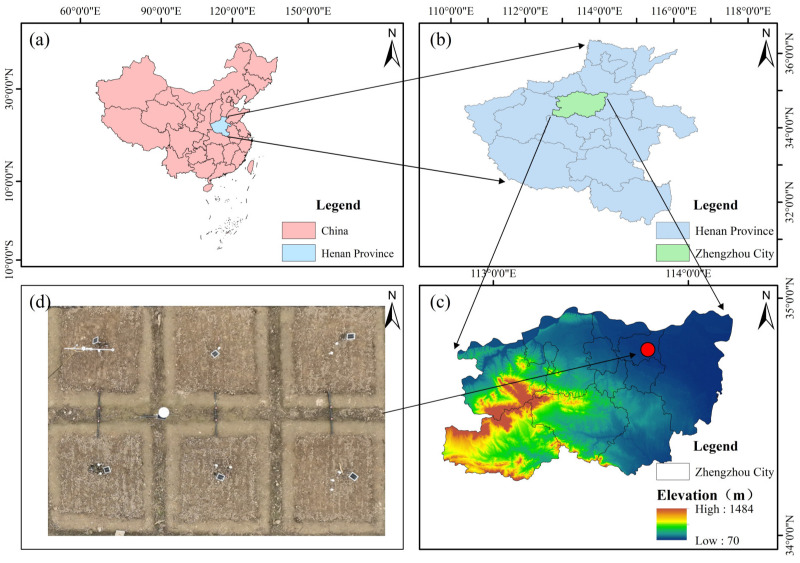
Location of the pilot zone. (**a**) China Map; (**b**)Henan Province map; (**c**) Elevation Map of Zhengzhou; (**d**) Overview of the Test Pit.

The experiment was conducted at the Experimental Field for Efficient Agricultural Water Use of North China University of Water Resources and Electric Power in Zhengzhou, Henan Province, China (34.75° N, 113.65° E, 110 m above sea level). The two-year field trial was performed from 2 June to 13 October 2024 and from 6 June to 30 September 2025. The location of the experimental site is illustrated in Figure 1. The site is characterized by a temperate continental monsoon climate, with a mean annual temperature of 14.3 °C and an annual sunshine duration of 2400 h. The average precipitation over the two-year study period was 1020.74 mm. The groundwater table at the experimental site was deeper than 15 m.

**Figure 2:**
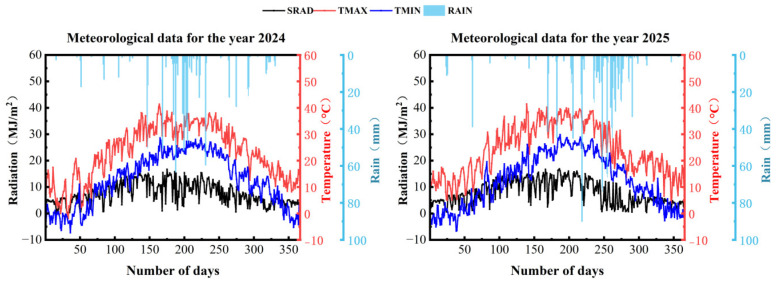
Meteorological data for the pilot zone.

Meteorological data were collected using a Tianqi Smart Ecological Weather Station (Model ET007, INSENTEK Crop., Hangzhou, China). The station continuously monitored air temperature, precipitation, wind speed, and sunshine duration, and the data were updated and uploaded at hourly intervals. The meteorological conditions during the two-year experimental period are shown in Figure 2. In 2024, the maximum temperature, minimum temperature, and mean temperature were 41.4 °C, −7.3 °C, and 17.86 °C, respectively. Precipitation was mainly concentrated in mid-July, with an annual total of 1034.31 mm. In 2025, the maximum temperature, minimum temperature, and mean temperature were 41.44 °C, −6.8 °C, and 18.32 °C, respectively. Precipitation was mainly concentrated in early August, with an annual total of 1007.16 mm.

### 2.2. Experimental Design

The maize variety used in this experiment was Shandong Denghai 605. The soil was classified as loam, and each experimental plot consisted of an open-air test pit measuring 3 m × 3 m. The average bulk density of soil at depths of 0–100 cm was 1.45 g/cm^3^, the field capacity (FC) was 25.91% (gravimetric water content), and the soil pH was 8.57. During the water treatment period, soil moisture in all treatments was maintained at 75–80% FC during the seedling stage and the non-drought irrigation stages. In the H treatment, soil moisture was maintained at 55–60% FC during the grain-filling stage to impose drought stress. In the Q treatment, soil moisture was maintained at 55–60% FC during the jointing stage to impose drought stress. In this study, 55–60% FC denotes water stress, and 75–80% FC indicates non-stress conditions. Urea (CH_4_N_2_O) was used as the nitrogen fertilizer, and three nitrogen application rates were established: low nitrogen (N3 = 100 kg·ha^−1^), normal nitrogen (N2 = 200 kg·ha^−1^), and high nitrogen (N1 = 300 kg·ha^−1^). During the growing season, nitrogen was applied in two split applications, with 50% applied before sowing and the remaining 50% top-dressed at the jointing stage. Different nitrogen application rates were combined with drought treatments imposed at different growth stages, resulting in six treatment combinations. Detailed descriptions of the six treatments are provided in Table 1.

300 kg·200 kg·100 kg·300 kg·200 kg·100 kg·Soil data were collected by an automated soil moisture monitoring system (Model ZL-06, INSENTEK Crop., Hangzhou, China), which continuously monitored soil moisture content. The sensor automatically recorded soil moisture at 60 min intervals at depths of 0–10 cm, 10–20 cm, 20–40 cm, 40–60 cm, 60–80 cm, and 80–100 cm. The basic physicochemical properties of each soil layer in the experimental area are presented in Table 2.

#### Methods for Monitoring Physiological Growth Indicators

(1) CWAM: At the maturity stage, five representative plants were sampled from each treatment pit. After removing adhering soil particles and weeds, the plants were separated into stems, leaves, and ears; the fresh weight of each component was measured separately. The samples were first heated at 105 °C for 30 min and then oven-dried at 75 °C to constant weight, after which the dry weight was recorded.

(2) Yield: At the maturity stage, five representative plants were selected from each treatment pit to determine yield components and ear traits, while the actual yield per pit was recorded separately. The measured variables included actual yield per pit (g/pit), 100-grain weight (g), ear weight (g), number of grains per ear, ear weight (g), ear length (cm), ear diameter (cm), number of rows per ear, and number of grains per row.

(3) LAI: Five representative plants with uniform growth were selected from each treatment pit. Leaf length and maximum leaf width were measured at 7 d intervals. The rectangular area of each leaf, calculated as leaf length × maximum leaf width, was multiplied by a correction factor of 0.75 to estimate leaf area [19]. Then, the mean value for the five plants was utilized as the leaf area of that pit. LAI was defined as the ratio of the total green leaf area per unit land area to the corresponding land area.

(1) $$\mathrm{LAI}=0.75\left(L\cdot W_{m}\right)/\left(S_{l}\cdot S_{r}\right)$$

where L denotes the leaf length, cm; $W_{m}$ represents the maximum leaf width, cm; $S_{l}$ and $S_{r}$ embody the row spacing and plant spacing in the test plot, respectively, cm.

(4) DMPEM:

(2) $$\mathrm{DMPEM}=\frac{\mathrm{DM}}{ET}$$

(3) ET=P+I−ΔW

where DM represents the total aboveground dry matter accumulation at maturity, kg·ha^−1^; ET signifies the total water consumption of maize throughout its growth cycle, mm; P denotes effective rainfall, mm; I embodies total irrigation, mm; ∆W refers to the change in soil water storage in the 0–100 cm soil layer, mm.

(5) DPNAM:

(4) $$\mathrm{DPNAM}=\frac{\mathrm{DM}}{N_{\mathrm{uptake}}}$$

(5) $$N_{uptake}=\sum_{i=1}^{n}\left(DM_{i}\times N_{i}\right)$$

where DM represents the total aboveground dry matter accumulation at maturity, kg·ha^−1^; $N_{uptake}$ embodies the total nitrogen uptake by the plant, kg·ha^−1^; ${DM}_{i}$ signifies the dry weight of each organ at maturity, kg·ha^−1^; $N_{i}$ indicates the corresponding total nitrogen content of each organ, %.

### 2.3. Model Performance

#### 2.3.1. DSSAT Model Data Processing

Cultivar parameters are among the most critical components in the DSSAT (v4.8) simulation process, and the accuracy of model outputs depends directly on the precision of their calibration. Existing approaches for determining cultivar parameters include manual and automatic parameter calibration. Manual calibration optimizes simulation performance step by step through iterative adjustment of variety parameters. In contrast, automatic calibration relies on a large number of database-driven simulations and generally provides higher precision. Common automatic calibration methods include Generalized Likelihood Uncertainty Estimation (GLUE), Markov Chain Monte Carlo (MCMC), and Particle Swarm Optimization (PSO). GLUE explores the parameter space through large-scale random sampling using the Monte Carlo method, identifies all ‘behaviorally acceptable’ parameter sets according to a predefined ‘likelihood function’, and then employs these parameter sets to characterize model uncertainty. MCMC is based on a Bayesian inference framework and explores the posterior probability distribution of parameters by constructing a Markov chain. In PSO, each ‘particle’ represents a parameter combination, and the parameter space is searched by tracking the individual best value (pbest) and the global best value (gbest).

SimLab software (v2.2), which is based on the principle of variance decomposition, integrates several well-established algorithms, including Sobol, Morris, and EFAST. It can capture interactions between parameters, rendering it suitable for sensitivity analysis of complex nonlinear models. First, the parameters used in this study are set in SimLab: thermal time from emergence to the end of the juvenile phase (P1); the critical photoperiod, defined as the longest day length (in hours) at which development proceeds at its maximum rate (P2); thermal time from silking to physiological maturity (P5); the maximum potential number of kernels per plant (G2); the kernel filling rate during the linear grain-filling stage under optimal conditions (G3); and the interval in thermal time (degree days) between successive leaf tip appearances (PHINT). Subsequently, Latin hypercubic sampling (LHS) was employed to generate 2000 sets of parameter combinations. Afterward, the parameter sets were input into the DSSAT model for batch simulation. Finally, the cultivar parameters were manually fine-tuned to derive calibrated values.

Following sensitivity and uncertainty analysis of the maize cultivar parameters, the calibrated parameter values are presented in Table 3.

Comprehensive analysis indicated that the above parameter values were suitable as maize variety parameters for this experiment and enabled accurate simulation of growth dynamics throughout the entire maize growth cycle.

#### 2.3.2. Model Validation

A statistical comparison between simulated and observed values of CWAM, Yield, LAI, DMPEM, and DPNAM was conducted to evaluate the accuracy of the DSSAT model under specific input conditions. Model performance was assessed using the coefficient of determination ($R^{2}$), root mean square error (RMSE), and normalized root mean square error (NRMSE).

(6) $$R^{2}=1-\frac{\sum_{i=1}^{n}{\left(M_{i}-S_{i}\right)}^{2}}{\sum_{i=1}^{n}{\left(M_{i}-\bar{M}\right)}^{2}}$$

(7) $$RMSE={\left[\frac{\sum_{i=1}^{n}{\left(M_{i}-S_{i}\right)}^{2}}{n}\right]}^{0.5}$$

(8) $$NRMSE=\frac{100}{\bar{M}}{\left[\frac{\sum_{i=1}^{n}{\left(M_{i}-S_{i}\right)}^{2}}{n}\right]}^{0.5}$$

where n denotes the sample size, $S_{i}$ denotes the simulated value; $\bar{S}$ represents the mean of the simulated values; $M_{i}$ signifies the measured value; $\bar{M}$ embodies the mean of the measured values. In this study, simulation accuracy is considered ‘excellent’ when $R^{2}$ is close to 1 and NRMSE ≤ 10%; when 10% ≤ NRMSE ≤ 20%, simulation accuracy is considered ‘good’ [20].

PCA, TOPSIS, and RSR were analyzed using R packages. The stats, FactoMineR, and psych packages were used for PCA; the rsr and epiDisplay packages were adopted for RSR. The TOPSIS entropy-weighting method can be implemented by the TOPSIS or MCDM packages, or calculated according to the following formula:

Common linear normalization:

(9) $$Z_{ij}=\frac{x_{ij}}{\sqrt{\sum_{i=1}^{n}{x_{ij}}^{2}}}$$

Calculate the positive and negative ideal solutions, where $Z^{+}$ denotes the most preferred option and $Z^{-}$ represents the least preferred option.

(10) $$Z^{+}=\left(\max z_{ij}\right)$$

(11) $$Z^{-}=\left(\min z_{ij}\right)$$

Calculate the distance to the positive and negative ideal solutions:

(12) $$D_{i}^{+}=\sqrt{\sum_{j=1}^{m}{\left(z_{ij}-z_{j}^{+}\right)}^{2}}$$

(13) $$D_{i}^{-}=\sqrt{\sum_{j=1}^{m}{\left(z_{ij}-z_{j}^{-}\right)}^{2}}$$

where $z_{ij}$ denotes the standardized value of the i evaluation object for the j indicator; $x_{ij}$ represents the raw data value of the i evaluation object for the j indicator.

The weight of the i-th object under the j-th metric is calculated by:

(14) $$p_{ij}=\frac{z_{ij}}{\sum_{i=1}^{m}z_{ij}}$$

The information entropy of the j-th metric is calculated by:

(15) $$E_{j}=-\frac{1}{\ln m}\sum_{i=1}^{m}p_{ij}\ln p_{ij}$$

The coefficient of variation for the j-th indicator is calculated by:

(16) $$d_{j}\;=\;1\;-\;E_{j}$$

The entropy weight for the j-th metric is calculated by:

(17) $$\omega_{j}=\frac{d_{j}}{\sum_{j=1}^{n}d_{j}}$$

where $E_{j}\in \left[0,1\right]$; a larger entropy value indicates smaller variability in the indicator and less information; m denotes the number of evaluation subjects; a larger weight $\omega_{j}$ reflects that the indicator is more crucial in the comprehensive evaluation.

The degree of closeness between the evaluation object for each year and the optimal scheme was calculated as the comprehensive evaluation score:

(18) $$C_{i}=\frac{D_{i}^{-}}{D_{i}^{-}+D_{i}^{+}}$$

where a higher value of $C_{i}$ indicates that the water and nitrogen management in year i is closer to the optimal level; the proximity coefficient $C_{i}$ ranges from 0 to 1; $C_{i}$ = 1 reflects that water and nitrogen utilization in that year is optimal.

## 3. Results

### 3.1. Results of Crop Growth Model Simulations

#### 3.1.1. Comparison of CWAM Simulated Values and Observed Values

**Figure 3:**
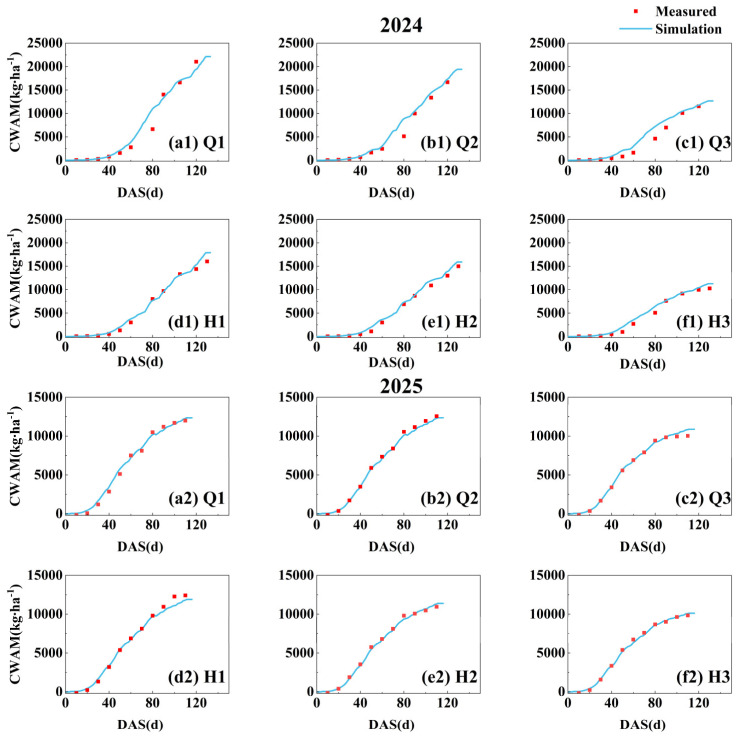
Comparison of CWAM output values with observed values under different processing methods.

As shown in Figure 3, the DSSAT model exhibited excellent simulation accuracy under different water–nitrogen coupling treatments. Throughout the growing season, the simulated and observed CWAM values showed highly consistent temporal patterns, characterized by a slow initial phase (DAS 0–30), rapid accumulation (DAS 30–90), and a steady decline (DAS 90–140). These results reveal that the model accurately captured the stage-specific dynamics of aboveground dry matter accumulation in summer maize. In both the H and Q groups, the simulated values clearly reproduced the gradient differences in CWAM among nitrogen application levels (N1 > N2 > N3). With the Q group as an example, the simulated maximum CWAM of the Q1 treatment reached 22,144 kg·ha^−1^ in 2024 and 12,352 kg·ha^−1^ in 2025, both of which were higher than those of the Q2 and Q3 treatments. Similarly, in the H group, the H1 treatment demonstrated the highest simulated CWAM within the group, reflecting that aboveground dry matter accumulation responded positively to increased nitrogen application. Overall, the CWAM values under the Q treatments were higher than those under the H treatments. Thus, drought during the jointing stage exerted a weaker inhibitory effect on CWAM than drought during the grain-filling stage.

#### 3.1.2. Comparative Analysis of Simulated Yield Values and Observed Data

**Figure 4:**
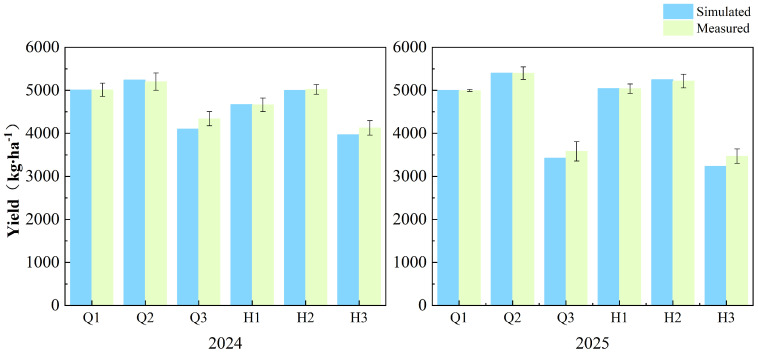
Comparison of simulated and observed yield values under different treatments.

As suggested in Figure 4, the yield trends predicted by the model were in close agreement with the field observations. In both cases, yield peaked under the N2 treatment and declined markedly under the N3 treatment, confirming the reliability of the model for analyzing water–nitrogen coupling effects. In both the Q and H groups, the simulated values accurately reproduced the yield gradient among nitrogen application levels (N2 > N1 > N3), consistent with the measured results. Additionally, the simulated and observed harvest index values exhibited highly similar variation patterns across all treatments, uncovering that the model accurately reflected the regulatory effects of drought stress and nitrogen metabolism on harvest index.

Over the two-year period, the simulated yield under the N1 treatment ranged from 4670 to 5040 kg·ha^−1^; that under the N2 treatment ranged from 5000 to 5400 kg·ha^−1^. Yield under the N1 treatment was slightly lower than that under the N2 treatment, indicating that although high nitrogen input can promote yield formation, it cannot fully offset the reduction in dry matter accumulation caused by water stress. Both the simulated and measured yields under the N2 treatment were the highest within each group. In other words, the normal nitrogen supply was more effective in balancing dry matter accumulation and the allocation efficiency of photosynthetic to grain. This ensures an adequate material basis for yield formation while avoiding the decline in harvest index associated with excessive nitrogen application. Yield under the N3 treatment was lower than that under the N2 treatment. Thus, under low-nitrogen conditions, nitrogen deficiency restricted the growth of vegetative organs, such as leaves and stems, reduced the proportion of photosynthates allocated to grain, and ultimately led to yield loss.

#### 3.1.3. Comparison of LAI Simulated and Observed Values

**Figure 5:**
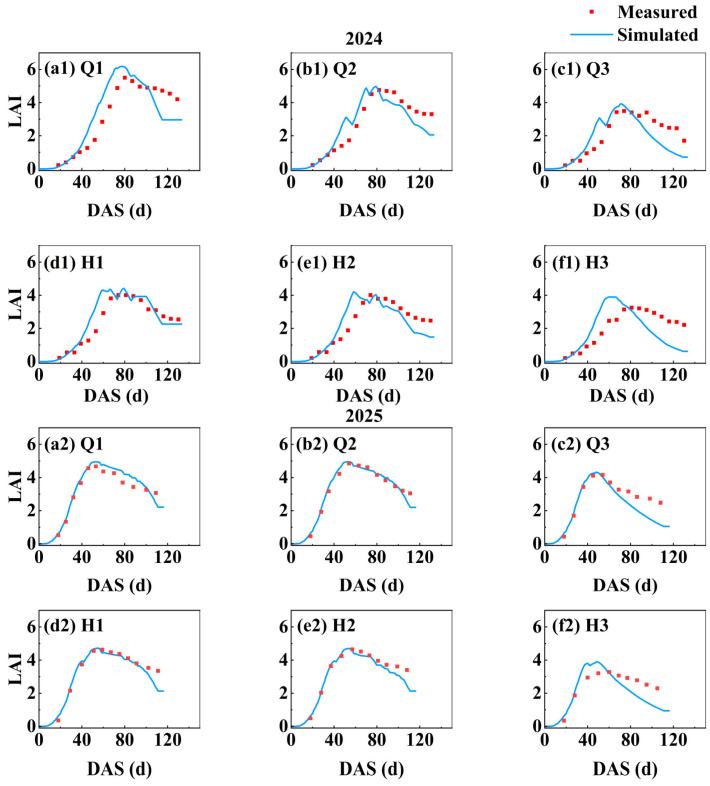
Comparison of simulated and measured LAI values under different treatment conditions.

As observed from Figure 5, the DSSAT model exhibited high simulation reliability and strong consistency in dynamic trends under different water–nitrogen coupling treatments. The simulated LAI dynamics were in close agreement with the field observations, with both showing a single-peaked curve characterized by a slow increase, a rapid rise to the peak, and a gradual decline. Hence, the model accurately captured the dynamics of leaf formation, expansion, and senescence throughout the maize growth period.

In both treatment groups, peak LAI increased with increasing nitrogen application rate. The simulated peak LAI ranged from 4.2 to 4.98 under the N2 treatment and from 4.42 to 6.19 under the N1 treatment. Although the peak LAI under the N2 treatment was slightly lower than that under the N1 treatment, the increase was more rapid, particularly between DAS 25 and 75. The subsequent decline was the slowest, with a gradual decrease after DAS 75. The simulated peak LAI under the N3 treatment was approximately 4, slightly lower than that under the N2 treatment, and fluctuations appeared during the later growth stage. For example, LAI in the Q3 treatment fluctuated after DAS 75, and the decline became more pronounced. This pattern reflects the dual effects of low nitrogen. While nitrogen deficiency inhibited leaf expansion and resulted in a lower peak LAI, reduced leaf photosynthetic performance during the later growth stage accelerated leaf senescence and decreased LAI stability.

#### 3.1.4. Comparative Analysis of DMPEM Simulated and Measured Values

**Figure 6:**
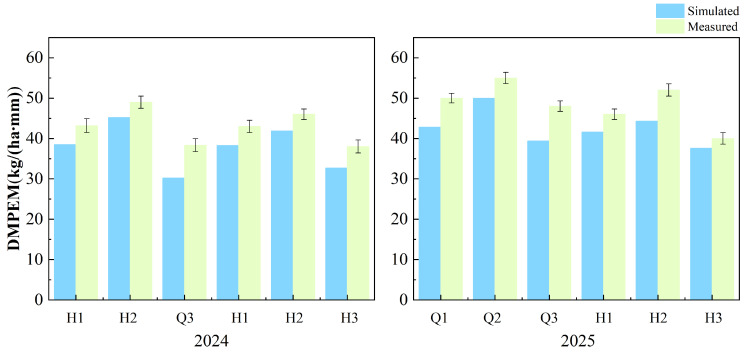
Comparison of simulated and measured DMPEM values under different processing conditions.

As reflected in Figure 6, DMPEM values in the Q group were generally higher than those in the H group, and the Q2 treatment showed the highest measured and simulated values over the two-year period. In both the Q and H groups, DMPEM followed the same pattern with increasing nitrogen application rate, namely N2 > N1 > N3. However, the simulated values were consistently lower than the measured values, highlighting an underestimation by the model of crop physiological responses under drought stress, such as stomatal regulation and nitrogen transport.

Under low-nitrogen conditions, the Q3 treatment consistently produced higher DMPEM than the H3 treatment, suggesting that the combined effect of drought and low nitrogen during the jointing stage increased DMPEM by suppressing transpiration water loss. Under high-nitrogen conditions, however, the expansion of leaf area enhanced photosynthetic capacity and increased transpiration demand. As a result, neither the Q nor the H treatments achieved the highest DMPEM under high nitrogen input.

#### 3.1.5. Comparative Analysis of Simulated and Measured DPNAM Values

**Figure 7:**
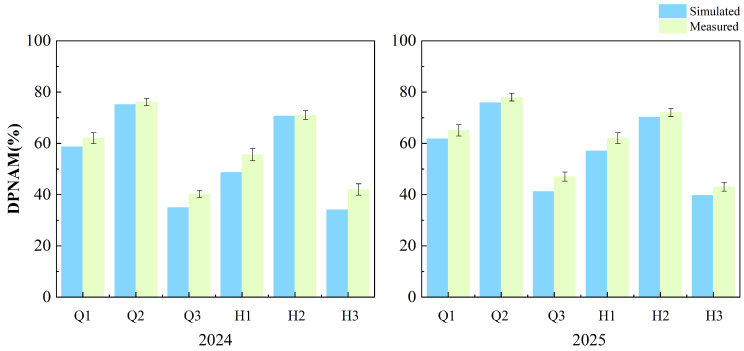
Comparison of simulated and measured DPNAM values under different processing conditions.

As revealed in Figure 7, DPNAM exhibited the same response pattern to nitrogen application rate in both the Q and H groups, with the order of N2 > N1 > N3. No significant difference was observed between the Q and H groups at the same nitrogen level.

The simulated and observed values demonstrated highly consistent trends, with similar gradient changes across nitrogen levels, further confirming the ability of the DSSAT model to analyze water–nitrogen coupling effects. For the Q2 treatment, the average deviation between simulated and measured DPNAM was 5–10% in both 2024 and 2025, suggesting relatively high simulation accuracy of the model under the combined N2 and Q treatment. DPNAM under the low-nitrogen treatment was significantly lower than that under the other treatments, and the difference between the Q and H groups was relatively small. In other words, nitrogen deficiency under drought stress substantially constrained DPNAM. Under the N1 treatment, DPNAM in Q1 was higher than that in H1 but lower than that under the N2 treatment. Thus, excessive nitrogen input led to nitrogen redundancy and thereby offset the positive effect of stage-specific drought regulation on DPNAM.

#### 3.1.6. Model Accuracy Evaluation

**Figure 8:**
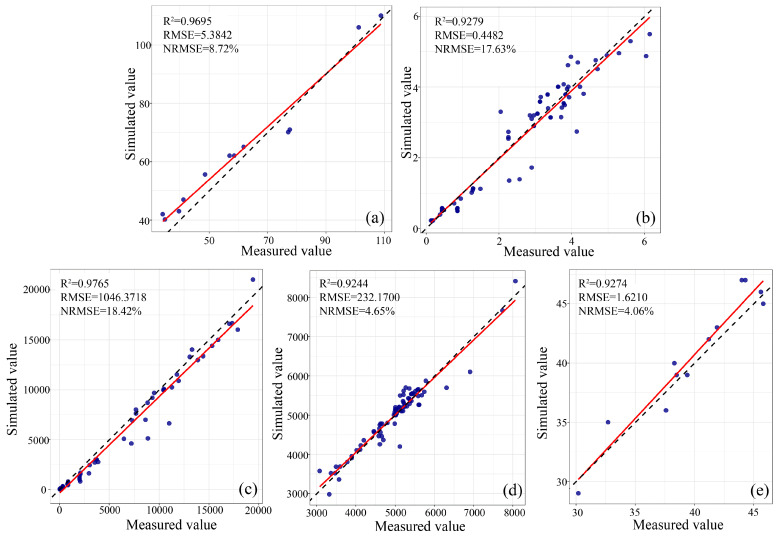
Analysis of the fit between measured and simulated values for each parameter. The blue dots represent points plotted with simulated and measured values as coordinates; The red straight line is the fitting line for all points; The black dashed line is a fixed 45-degree diagonal for reference only. (**a**) DPNAM fit; (**b**) LAI fit; (**c**) CWAM fit; (**d**) Yield fit; (**e**) DMPEM fit.

Figure 8 presents a comparative analysis of simulated and observed values for CWAM, Yield, LAI, DMPEM, and DPNAM, with $R^{2}$ ranging from 0.9244 to 0.9765 and NRMSE ranging from 4.06% to 18.42%. The RMSE was 1046.3718 for CWAM, 232.17 for yield, 0.4482 for LAI, 1.621 for DMPEM, and 5.3842 for DPNAM. These results imply that the model achieved high simulation accuracy and overall good performance.

### 3.2. Assessment of Water–Nitrogen Coupling Management

**Figure 9:**
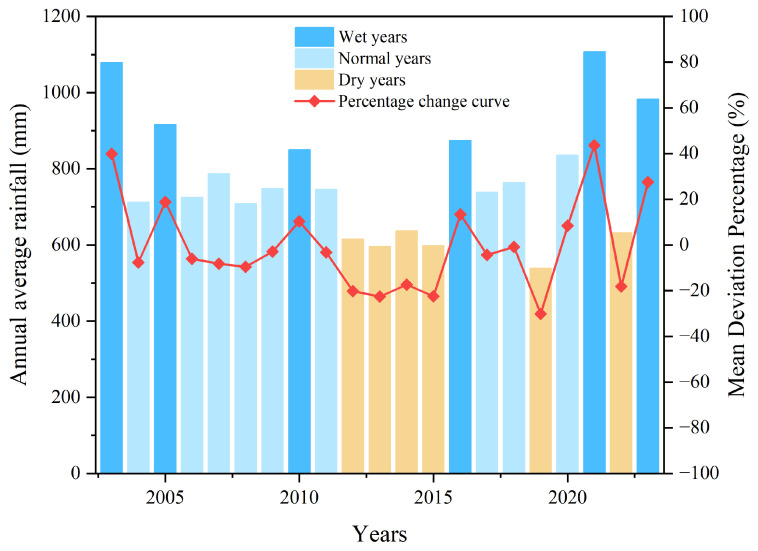
Classification by level and year type.

Following calibration and validation of the DSSAT model parameters using different treatments from the 2024 and 2025 summer maize field experiments, data for Henan Province from 2003 to 2023 were incorporated into the model for comprehensive evaluation. According to an average annual precipitation of 771.07 mm, the years were divided into three categories: wet years (annual rainfall ≥ 110% of the average), normal years (annual rainfall ranging from 90% to 110% of the average), and dry years (annual rainfall ≤ 90% of the average) (Figure 9). Subsequently, a comprehensive evaluation was performed.

#### 3.2.1. Output Data and Analysis for Different Hydrological Years

Table 4 suggests that the meteorological data from 2003 to 2023 were classified into dry, normal, and wet years according to rainfall frequency. The mean values of the indicators for the three year types were utilized as the raw data for the comprehensive entropy-weighted analysis.

Based on the observed and model-simulated data, the raw values of five indicators under six treatments were compiled. Outlier testing was performed using Grubbs’ test (*p* > 0.05) to confirm the absence of extreme values. Additionally, coefficient of variation analysis (CV < 15%) was conducted to verify the stability of replicate data within each treatment, laying a reliable data foundation for subsequent entropy-weighting calculations.

#### 3.2.2. PCA

The variable loadings plot is one of the most fundamental visualization tools in PCA, intuitively representing the distribution of original variables in the principal component space after dimensionality reduction. Its main purpose is to reveal the correlations among variables, their contributions to the principal components, and the discriminating ability of each variable in the reduced-dimensional space.

**Figure 10:**
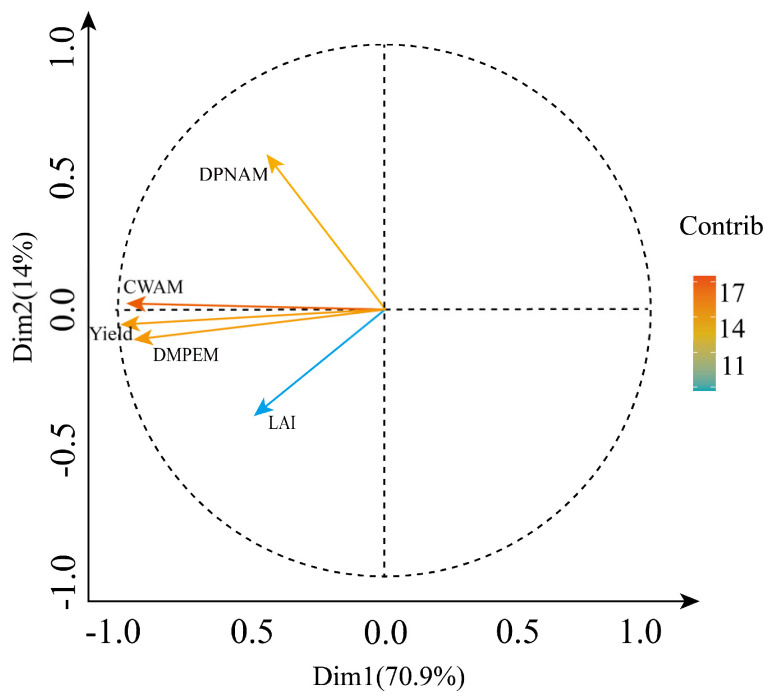
PCA variable contribution plot.

Figure 10 and Table 5 show that the first two principal components explained 84.9% of the total variance, indicating that they effectively captured most of the information contained in the original dataset. Dim1, which accounted for 70.9% of the variance, was the dominant principal component, with all variables distributed along the negative X-axis. Among them, Yield, DMPEM, and CWAM were the most influential variables, exhibiting the highest loadings (close to −1). These variables were strongly positively correlated with one another and strongly negatively correlated with Dim1. In contrast, the loadings of DPNAM and LAI were much smaller and nearly indistinguishable, implying weak correlations between these variables and a relatively weak negative correlation with the principal component. In Dim2, which explained 14% of the variance, LAI, Yield, DMPEM, and CWAM were the main positively loaded variables and demonstrated strong positive correlations with one another. However, CWAM had the smallest loading, close to 0, reflecting the weakest positive correlation with this principal component. DPNAM is a negative core variable, which has weak correlations with other variables and a strong negative correlation with the principal component.

Overall, the Yield, DMPEM, and CWAM vectors demonstrate a high degree of overlap and a positive correlation (Pearson correlation coefficients all > 0.92, *p* < 0.05), reflecting the synchrony between yield increases and biomass accumulation as well as efficient water use. Furthermore, Yield is the most critical target variable in agricultural production. Therefore, only Yield is selected for subsequent analysis. DPNAM has the highest combined loadings across the two principal components and features strong correlations in both, while revealing weak correlations with other variables. In other words, it provides independent information. Consequently, this indicator is retained for further analysis.

#### 3.2.3. Entropy-Weighted Analysis Using the TOPSIS Method

Based on the PCA results, a comprehensive evaluation was further conducted by the TOPSIS method. This approach identifies the overall performance of each treatment by constructing positive and negative ideal solutions and calculating the relative distances of each evaluation object from the optimal and worst scenarios.

For the purpose of avoiding biases resulting from subjective weighting, the entropy weighting method was employed to objectively determine the weights of each evaluation indicator. The weights for each indicator were calculated based on the CWAM, Yield, LAI, DMPEM, and DPNAM data in Table 4, and the results are detailed in Table 6.

The comprehensive performance of the different water and fertilizer regimes was ranked according to the mean TOPSIS proximity coefficient. As observed from Table 7, the mean proximity values ranged from 0.369 to 0.629, revealing clear differences among the water and fertilizer regimes in their overall effects on maize performance. Among all treatments, Q2 had the highest mean proximity value and ranked first, reflecting the best comprehensive performance. H2 ranked second, whereas Q3 had the lowest mean proximity value and ranked sixth, suggesting the poorest overall performance. To sum up, the Q2 and H2 treatments maintained relatively high overall benefits under different hydrological conditions, and therefore represented the most stable and advantageous water and fertilizer management regimes in this study. The final ranking of the six water and fertilizer regimes was determined entirely by the mean TOPSIS proximity coefficient; a higher mean value indicates better overall performance in water and fertilizer use efficiency, crop growth, and yield across multiple years.

#### 3.2.4. RSR Analysis

This study employed the Relative Rank Sum (RSR) method for multi-criteria evaluation. Rank transformation is a key preliminary step in the RSR method, as it converts raw indicator values with different dimensions and directions into a unified ranking scale. Hence, it eliminates dimensional interference and inconsistencies in indicator direction, enabling the objective integration of multiple indicators.

As observed from Table 8, for benefit-type indicators, higher raw values were assigned higher ranks, indicating better performance. The raw values of the 18 evaluation objects were ranked in descending order, with the highest value assigned a rank of 18 and the lowest assigned a rank of 1. When identical raw values occurred, the average rank was assigned. This procedure ultimately generated a rank matrix based on five indicators.

**Figure 11:**
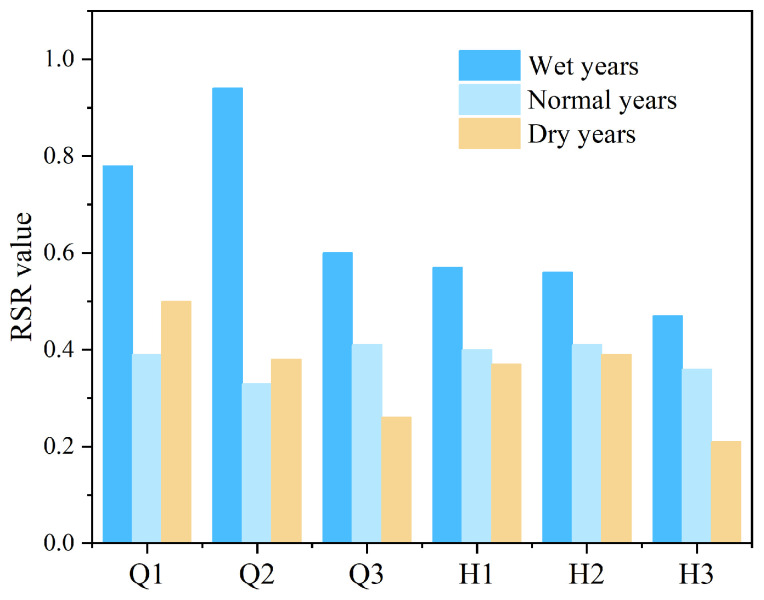
RSR composite score.

Figure 11 reveals that the RSR values of the 18 treatment-year combinations ranged from 0.211 to 0.944, with substantial overall variation. In other words, different water and fertilizer management regimes, together with hydrological year conditions, had marked effects on the overall growth performance and water–fertilizer use efficiency of maize. The closer the RSR value is to 1, the better the overall performance of the corresponding treatment. Across different hydrological year types, the RSR values in wet years were generally higher than those in normal and dry years. Specifically, the RSR values ranged from 0.467 to 0.944 in wet years, with a mean of 0.651; from 0.333 to 0.411 in normal years, with a mean of 0.385; and from 0.211 to 0.550 in dry years, with a mean of 0.357. These results specify that water availability was the key environmental factor regulating the overall performance of maize. The superior overall performance observed in wet years further suggests that water supply plays a central role in regulating maize growth and resource use efficiency. Among all treatment–year combinations, the Q2 treatment in wet years had the highest RSR value (0.944), making it the optimal treatment overall. This result reflects a high degree of synergy among yield, dry matter accumulation, and water–fertilizer use efficiency. The Q1 treatment in wet years ranked second, with an RSR value of 0.778, and represented a favorable regime with potential for both high yield and water saving. In dry years, the Q3 and H3 treatments had the lowest RSR values and the poorest overall performance, underscoring insufficient tolerance to water stress and inadequate matching between water and fertilizer inputs. These findings provide a scientific basis for selecting optimal water and fertilizer management regimes under different hydrological conditions in the study area.

## 4. Discussion

### 4.1. The Effects of Water and Nitrogen Inputs on Yield, DMPEM and DPNAM

Water is the primary limiting factor for crop growth. When rainfall is insufficient to meet crop water demand, supplementary irrigation is required to maintain favorable growth conditions [21], particularly in rainfed agricultural regions. The effects of water stress vary significantly across crop growth stages. Nitrogen, as an essential nutrient, plays a decisive role in determining yield potential [22]. The experimental results suggested that under drought stress imposed at different growth stages, the simulated and measured values of Yield, DMPEM, and DPNAM first increased and then decreased with rising nitrogen application rate, reaching their highest levels under the N_2_ treatment. This finding is consistent with the global meta-analysis of Li et al. (2019) [1]. Specifically, moderate nitrogen fertilization can synergistically improve yield, DMPEM, and DPNAM, whereas excessive or insufficient nitrogen supply reduces nitrogen-sensitive productivity and efficiency indicators [23]. Specifically, an appropriate nitrogen supply promotes root development and grain accumulation [3] while avoiding the resource waste and yield reduction associated with excessive nitrogen input under the N1 treatment [24]. Notably, although LAI was highest under the N1 treatment, yield did not increase accordingly. This implies that under water-limited conditions, excessive nitrogen input cannot be effectively converted into grain yield and may even aggravate transpirational water loss, thereby reducing water use efficiency [7,25]. Additionally, DMPEM was generally higher in the Q group than in the H group, and both the measured and simulated values were highest under the Q2 treatment. Thus, moderate water deficit during the jointing stage can improve water use efficiency per unit water consumed by optimizing stomatal regulation [26,27]. After rewatering, crop water use and photosynthetic capacity were fully restored, which enhanced subsequent tolerance to water stress and enabled the crop to maintain high photosynthetic rates and dry matter allocation capacity during later growth stages [27,28]. Chen et al. (2023) [29] discovered that maize subjected to mild drought stress during the jointing stage can promote root development and aboveground growth while effectively improving the plant’s water use efficiency. In contrast, drought during the grain-filling stage brings about an irreversible decline in photosynthetic performance, which is difficult to fully recover even after rewatering, and exerts limited effects on improving DMPEM [30]. For DPNAM, the N_2_ treatment produced the highest values, and no significant difference was observed between the Q and H groups at the same nitrogen level. In other words, DPNAM was primarily regulated by nitrogen application rate. This is consistent with the findings of Li et al. (2025) [31]. Although total nitrogen content increased under high-nitrogen conditions, nitrogen utilization within the plant may have been constrained [32], resulting in reduced DPNAM. Under low-nitrogen conditions, nitrogen becomes the dominant limiting factor, severely inhibiting photosynthesis and accelerating leaf senescence, which in turn leads to a marked decline in DPNAM [33]. According to Arisede et al. (2020) [34], changes in DPNAM are more pronounced under low nitrogen input than under high nitrogen input, because nitrogen limitation has a greater effect than utilization efficiency when nitrogen uptake is not restricted.

### 4.2. The Effects of Water and Nitrogen Inputs on CWAM and LAI

In summer maize production, CWAM and LAI are key parameters. As quantitative indicators of plant architecture, they reflect crop growth status and indirectly affect crop performance [35]. The experimental results established that the simulated values of CWAM and LAI were highly consistent with the measured values. Hence, the DSSAT model accurately captured the contribution of the photosynthetic system to both LAI and CWAM [36,37]. Under drought stress imposed at different growth stages, the highest CWAM and LAI values were observed under the N1 treatment as nitrogen application increased, consistent with the findings of Shou et al. (2023) [38] and Gao et al. (2024) [39]. Adequate nitrogen supply is fundamental to chlorophyll synthesis and photosynthesis. As nitrogen input increases, leaf expansion accelerates, LAI rises, and light interception efficiency is consequently improved, laying the physiological foundation for dry matter accumulation [40]. Nonetheless, although the N1 treatment maximally stimulated leaf expansion, the resulting excessive vegetative growth could not be fully translated into a yield advantage under water stress [41]. This finding is consistent with the view proposed by Hernández et al. (2021) [25] that high nitrogen increases transpiration but does not necessarily increase yield. Although the peak LAI under the N2 treatment was slightly lower than that under N1, its decline was slower, allowing photosynthesis to be sustained for a longer period. Under the N3 treatment, both CWAM and LAI were at their lowest levels, and their growth rates lagged behind those of the other treatments, reflecting the severe restriction of photosynthesis and chlorophyll function caused by nitrogen deficiency [42]. Both peak CWAM and peak LAI were higher in the Q group than in the H group, revealing that summer maize was more sensitive to water conditions during the grain-filling stage in the later growth period. Water stress during this stage severely restricted photosynthate production and grain filling [43]. This result is similar to the findings of Rugira et al. (2021) [44]. However, the present findings differ from those reported by Dou et al. (2024) [45], who reported that drought during the grain-filling stage primarily affects grain filling, with only a limited direct effect on LAI. This discrepancy may be related to differences in sowing time and soil characteristics, which can influence crop responses to water stress.

### 4.3. Assessment Across Different Hydrological Years

The long-term DSSAT simulation based on meteorological data from 2003 to 2023, combined with the integrated PCA-TOPSIS-RSR evaluation framework, enables a more objective assessment of the reliability of different water and nitrogen management strategies under various hydrological year types. Water supply is a key factor regulating maize growth, development, and resource use efficiency [46,47,48]. In wet years, abundant precipitation substantially improves the crop growth environment. As a result, RSR values are markedly higher than those in normal and dry years. Particularly, the Q2 treatment achieved an RSR value of 0.944, reflecting that under conditions of ample precipitation, the combination of moderate drought at the jointing stage and normal nitrogen application can maximize high and stable yields [3]. Nevertheless, the Q2 treatment also exhibited a relatively large standard deviation, implying weak interannual stability and greater sensitivity to water fluctuations. In contrast, the overall performance of the H2 treatment was second only to that of Q2, while exhibiting better interannual stability. Therefore, in practical production, the Q2 treatment may be selected to pursue maximum yield when weather predict a humid hydrological year. When interannual precipitation is unstable, however, the Q1 or H2 treatments may be preferable, as they maintain a better balance between high yield and yield stability across different hydrological year types, making them more suitable water and fertilizer management regimes for regional promotion [49].

### 4.4. Limitations and Future Research Directions

This study still has several limitations. First, the experimental period was relatively short. Although rainfall conditions significantly differ between the two years and represent different types of growing seasons, maize growth is influenced by the combined effects of multiple factors. Second, while the DSSAT model has been widely used to assess the impacts of climate change on crops, it may not have fully accounted for all biotic and abiotic factors affecting crop growth and yield during the experimental period, which could have contributed to discrepancies between the simulated results and field observations [50,51]. Finally, the statistical evaluation metrics were mainly based on physiological and growth-related indicators, such as yield and cumulative aboveground dry matter, and did not incorporate economic costs and benefits. Notably, the Q2 and H2 treatments performed well overall in this study. Their nitrogen application rates were lower than those of the high-nitrogen treatment, thereby correspondingly reducing nitrogen fertilizer costs and offering certain economic savings potential. Hence, the optimal management strategies identified in this study still should be further evaluated before practical application.

Future research should expand both the spatial and temporal scope of the trials to obtain more comprehensive datasets, so as to improve the accuracy and spatiotemporal representativeness of the model parameters. By integrating the calibrated DSSAT model with local weather forecasts and soil moisture monitoring data, an online decision support system could be developed for individual farmers or agricultural management departments to provide recommendations on optimal management strategies. Additionally, economic and environmental indicators should be incorporated alongside agronomic metrics in future evaluations to identify optimal management strategies that achieve high efficiency, high yield, and low cost.

## 5. Conclusions

This study adopted the DSSAT model to simulate and evaluate the results of two years of field experiments under different hydrological year types. The main conclusions are drawn as follows:

(1) Nitrogen application rate exerted a significant regulatory effect on yield, DPNAM, and DMPEM. All three variables first increased and then decreased with rising nitrogen application rate, while the optimal values were observed under the normal nitrogen treatment. Drought imposed during the jointing stage and grain-filling stage had relatively similar effects on yield, DPNAM, and DMPEM. Under the high-nitrogen treatment, LAI and CWAM reached their maximum values. Compared with drought during the grain-filling stage, drought during the jointing stage presented a smaller negative effect on CWAM and LAI.

(2) Drought during the jointing stage combined with normal nitrogen application is the optimal water–nitrogen management strategy in wet years. Drought during the jointing stage combined with high nitrogen application, as well as drought during the grain-filling stage combined with normal nitrogen application, demonstrates greater adaptability and broader applicability across different hydrological year types.

## Figures and Tables

**Table 1:** Different water-holding capacities and nitrogen application rates in the experimental plots.

Treatments	Fertilizer Management	Water Management (Target Soil Moisture as % Field Capacity)	
		Jointing Stage	Grain-Filling Stage
Drought and high nitrogen levels during the jointing stage (Q1)	300 kg·ha^−1^	55–60% FC	75–80% FC
Drought during the jointing stage and normal nitrogen levels (Q2)	200 kg·ha^−1^		
Drought and low nitrogen during the jointing stage (Q3)	100 kg·ha^−1^		
Drought and high nitrogen levels during the grain-filling stage (H1)	300 kg·ha^−1^	75–80% FC	55–60% FC
Drought during the grain-filling stage and normal nitrogen levels (H2)	200 kg·ha^−1^		
Drought and low nitrogen during the grain-filling stage (H3)	100 kg·ha^−1^		

**Table 2:** Data on the physical and chemical properties of the soil in the pilot area.

Soil Depth (cm)	0–20	20–40	40–60	60–80	80–100
Bulk density, moist (g·cm^−3^)	1.46	1.47	1.47	1.48	1.47
$\mathrm{Drained}\;\mathrm{upper}\;\mathrm{limit}\;({\mathrm{cm}}^{3}\cdot$cm^−3^)	0.34	0.32	0.27	0.31	0.28
pH in buffer	8.47	8.52	8.68	8.62	8.80
$\mathrm{The}\;\mathrm{lower}\;\mathrm{limit}\;\mathrm{of}\;\mathrm{plant}\;\mathrm{extractable}\;\mathrm{soil}\;\mathrm{water}\;({\mathrm{cm}}^{3}\cdot$cm^−3^)	0.15	0.14	0.15	0.11	0.12
$\mathrm{The}\;\mathrm{upper}\;\mathrm{limit},\;\mathrm{saturated}\;({\mathrm{cm}}^{3}\cdot$cm^−3^)	0.41	0.40	0.37	0.40	0.41
Sat. hydraulic conductivity (cm·h^−1^)	2.33	1.66	1.83	1.83	2.19

**Table 3:** Cultivar parameters.

Parameter Name	P1	P2	P5	G2	G3	PHINT
Parameter values	350.8	0.633	900	437.30	15.84	45.90

**Table 4:** Data on growth and physiological indicators for different hydrological years.

Category	Processing	CWAM (kg·ha^−1^)	Yield (kg·ha^−1^)	LAI	DMPEM (kg/(ha·mm))	DPNAM (%)
Wet years	H1	16,201	5003.75	1.9	39.8	27.17
	H2	15,892	4368.25	2	46.2	47.25
	H3	14,521	4113	1.5	35.3	72.1
	Q1	16,025	5112.5	3	49.4	33.6
	Q2	15,725	5271.75	2.9	59.7	61
	Q3	13,748	4633.5	2	38.9	79.45
Normal years	H1	6013	2927	2.5	27	30
	H2	6340	3266	2.4	28.4	21
	H3	5246	2269	2.3	23.3	52.5
	Q1	6016	2947	2.5	26.9	30
	Q2	6291	3259	2.4	28.1	21
	Q3	5318	2325	2.3	23.6	53
Dry years	H1	4659	2604	1.2	27.7	19.5
	H2	4849	2774	1.5	28.5	13.7
	H3	4222	2153	1.2	24.8	34.1
	Q1	4675	2621	1.6	27.5	19.5
	Q2	4880	2792	1.6	28.3	13.7
	Q3	4190	2502	1.5	25.3	34.5

**Table 5:** PCA Variance and Eigenvalues.

Principal Component	Variance Ratio (%)	Eigenvalue
Dim1	70.9	3.545
Dim2	14.0	0.700

**Table 6:** Weights of each evaluation criterion.

Evaluation Criteria	Weights
CWAM	0.18
Yield	0.26
LAI	0.12
DMPEM	0.22
DPNAM	0.22

**Table 7:** Ranking of final fit.

Sort	Processing	Mean Proximity	Standard Deviation of Proximity	Standard Error
1	Q2	0.629	0.302	0.174
2	H2	0.571	0.218	0.126
3	Q1	0.560	0.203	0.117
4	H1	0.429	0.192	0.111
5	H3	0.396	0.256	0.148
6	Q3	0.369	0.281	0.162

**Table 8:** RSR rank-order transformation data.

Year	Processing	Yield Rank	LAI Rank	DMPEM Rank	DPNAM Rank	CWAM Rank
Wet years	H1	15	6	10	5	15
	H2	10	7	13	12	8
	H3	9	3	7	17	6
	Q1	16	18	15	8	13
	Q2	18	17	18	14	18
	Q3	11	7	9	18	9
Normal years	H1	6	16	4	6	4
	H2	8	14	6	2	7
	H3	3	12	1	13	3
	Q1	5	16	3	6	5
	Q2	7	14	5	2	2
	Q3	4	12	2	15	4
Dry years	H1	11	1	14	4	3
	H2	14	3	16	1	1
	H3	1	1	7	9	1
	Q1	12	6	12	4	11
	Q2	13	6	11	1	3
	Q3	2	3	8	9	1

## Data Availability

Data and materials are available upon request from the corresponding authors. We only provide the original data to applicants who have reasonable scientific research. This is because the authors have the right to retain the original data to prevent situations where the original data of this study is used without informing the author.
